# Three New Cycloartenol Triterpenoid Saponins from the Roots of *Cimicifuga simplex* Wormsk

**DOI:** 10.3390/molecules16064348

**Published:** 2011-05-25

**Authors:** Haixue Kuang, Yang Su, Bingyou Yang, Yonggang Xia, Qiuhong Wang, Zhibin Wang, Zhengfan Yu

**Affiliations:** Key Laboratory of Chinese Materia Medica, Heilongjiang University of Chinese Medicine, Ministry of Education, Harbin 150040, China

**Keywords:** *Cimicifuga simplex* Wormsk, 9,19-cyclanstane triterpenic glycosides, cycloartenol triterpenoid saponins

## Abstract

Three new cycloartenol triterpene saponins, named shengmaxinsides A-C, have been isolated from the ethyl acetate soluble fraction of an ethanol extract of *Cimicifuga simplex* Wormsk roots. Their structures were established by chemical tests and detailed spectroscopic analysis as 25-*O*-acetyl-7,8-didehydrocimigenol-3-*O-β*-D-galactopyranoside (**1**), 7,8-didehydrocimigenol-3-*O-β*-D-galactopyranoside (**2**) and 7,8-didehydro-24*S*-*O*-acetylhydroshengmanol-3-*O-β*-D-galactopyranoside (**3**), respectively.

## 1. Introduction

**Figure 1:**
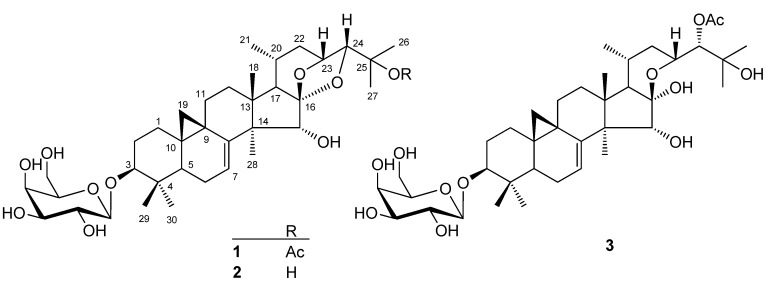
Structures of **1-3**.

The Ranunculaceae is a small family with five genera and around 19 species found throughout the World. Currently, about nine Cimicifuga species grow in China. *C.* *simplex* (Shengma in Chinese) is a deciduous perennial herb widely distributed in China. Traditionally, the root of *C.* *simplex* has been used in oriental countries as an anti-inflammatory and anti-viral agent [1,2,3] and the beneficial ingredients responsible for the anti-inflammatory effects are ferulic acid and isoferulic acid [4,5]. This herb has also been used for the treatment of human immunodeficiency virus (HIV), and its more general analgesic, antipyretic, antidiabetes, antimalaria and vasoactive properties [3,4,5]. Its chemical constituents have been extensively investigated and the main constituents are 9,19-cyclolanostane triterpenoid glycosides, flavonoids, alkaloids, and chromones [6,7,8,9]. More than 200 uncommon cycloartane-type triterpenoid saponins have been isolated from Cimicifuga plants [6]. Genjiro and his team have isolated more than fifty cycloartane-type triterpenoids from *C. simplex* grown in Japan [10,11,12,13,14,15,16,17,18,19,20,21,22]. It was reported that 9,19-cyclolanostane triterpene glycosides exhibited antiosteoporosis, anti-tumor and anti-complement activities [23,24,25]. Furthermore, triterpenoids may be useful candidates for the development of new drugs for cardiovascular disorders due to their antioxidant and anti-inflammatory activity [3]. In continuation of our search for pharmacological and structurally interesting substances from Chinese traditional herbal drugs, we investigated the chemical constituents of *C.* *simplex*. Fractionation of the ethyl acetate soluble extract of the roots of *C.* *simplex* by column chromatography afforded three new cycloartane-type triterpenoid saponins (Figure 1). We report here on the isolation and structural elucidation of these compounds by chemical and spectroscopic analysis.

## 2. Results and Discussion

Compound **1**, named shengmaxinside A, was obtained as colorless needles and gave positive results for the *Liebermann-Burchard* reaction and *Molish* reagents, indicating it to be a triterpenoid glycoside. Its molecular formula was established as C_38_H_58_O_11_ by the positive HRESIMS from the [M-H_2_O+H]^+^ and [M+Na]^+^ signals at *m/z* 673.3964 (calc. for C_38_H_57_O_10_, 673.3952) and 713.3891 (calc. for C_38_H_58_O_11_Na, 713.3877), respectively, indicating ten degrees of unsaturation.

The ^1^H-NMR spectrum (Table 1) showed the presence of cyclopropane methylene groups at *δ*_H_ 0.47 and 1.06 (each 1H, *d*, *J =* 3.6 Hz), six tertiary methyl groups at *δ*_H_ 1.04, 1.17, 1.33, 1.47, 1.53 and 1.64, a secondary methyl at *δ*_H_ 0.96 (1H, *d*, *J =* 5.6 Hz), an acetyl methyl at *δ*_H_ 2.01, one anomeric proton at *δ*_H_ 4.89 (1H, *d*, *J =* 7.6 Hz), four oxygenated methine protons (*δ*_H_ 3.51, 3.77, 4.53, 4.60) and a series of overlapped signals suggesting a cycloartane-type triterpene glycoside. The ^13^C-NMR spectrum (Table 1) displayed a total of thirty eight carbon signals due to the aglycon moiety, along with a sugar unit and an acetyl unit. The ^13^C-NMR spectrum exhibited anomeric carbons at *δ*_C_ 107.5. All the above evidence suggested that **1** was a highly oxygenated 9,19-cycloartane triterpene glycoside. Moreover, *δ*_C_ 112.8 suggests **1** to be a cimigenol type saponin [26].

After acid hydrolysis and derivatization as alditol acetates, the gas chromatography (GC) analysis revealed the presence of D-galacose. The presence of a galacose was further confirmed by its NMR data [16], and the galactose linkage was assigned as *β* from observation of the anomeric proton coupling constant at *δ*_H_ 4.89 (1H, *d*, *J =* 7.6 Hz). The residual three further signals at *δ*_H_ 4.60 (1H, *ddd*, *J =* 2.0, 4.3, 9.2 Hz), 4.53 (1H, *d*, *J =* 7.6 Hz) and 3.77 (1H, *d*, *J =* 4.4 Hz) in the region of aglycon moiety suggest three additional oxygen-bearing carbons on the aglycone. This hypothesis was confirmed by the HMBC spectrum, which showed cross-peaks between proton signal at *δ*_H_ 4.53 (1H,*d*, *J =* 7.6 Hz) with C-14 and C-16, C-13 and C-17, between proton signal at *δ*_H_ 4.60 (1H, *ddd*, *J =* 2.0, 4.3, 9.2 Hz) with C-24 and C-22, and between proton signal at *δ*_H_ 3.77 (1H, *d*, *J =* 4.4 Hz) with C-23 and C-25. This unambiguously iindicated that the oxygen-bearing carbons are C-15, C-23 and C-24. In the HMBC spectrum, significant correlations between *δ*_H_ 4.89(H-1') and 88.4(C-3) suggested that the galactopyranosyl was located at the C-3 position. Furthermore, the long-range correlations between an acetyl proton (*δ*_H_ 2.01) with C-25 (*δ*_C_ 79.8) indicated that the acetyl unit locating at C-25. Other key long-range correlations were observed for H-19/C-19, H-1'/C-3, H-24/C-25, H-23/C-22 and C-24, and an acetyl methyl proton and an acetyl carbon and C-25. 

Comparison of the ^13^C-NMR spectral data of **1** with those of the known compound 25-*O*-acetyl-7,8-didehydrocimigenol-3-*O-β*-D-xyloside [19] showed that the aglycone of **1** was very similar to that of the known compound, except for the signals of the sugar moieties. This suggested that **1** had the same aglycone as 25-*O*-acetyl-7,8-didehydrocimigenol-3-*O-β*-D-xyloside. Thus, from the above the ^1^H -^1^H COSY, HSQC, DEPT and HMBC we concluded that the planar structure of **1** corresponded to 25-*O*-acetylcycloartane-7-en-3-*O-β*-D-galactopyranoside.

Compound **2**, named shengmaxinside B, was obtained as colorless needles and gave positive results for the *Liebermann-Burchard* reaction and *Molish* reagent, which was considered evidence of a triterpenoid glycoside. Its molecular formula was established as C_36_H_56_O_10_ by the positive HRESIMS from the [M-H_2_O+H]^+^ and [M+Na]^+^ signals at *m/z* 631.3884 (calc. for C_36_H_55_O_9_ 631.3846) and 671.3802 (calc. for C_36_H_56_O_10_Na 671.3771), respectively, indicating nine degrees of unsaturation.

In the ^13^C-NMR spectrum (Table 1) a total of thirty six carbon signals due to the aglycon moiety were observed, along with a sugar unit. Compared to **1**, there is no acetyl unit signal. In the meantime, only the chemical shifts of C-25, C-26 and C-27, located at *δ*_C_ 68.6, 30.7, 25.9, respectively, were changed compared to **1**. The comparison of the ^13^C-NMR data of **2** to those of the moieties of the ether-linkage and ester-linkage sugar chains of **1** suggested that **2** possessed the same sugar chains as **1**. This deduction was confirmed by the HMBC experiment. On the basis of these data, **2** was elucidated as 7,8-didehydrocimigenol-3-*O-β*-D-galactopyranoside. 

Compound **3**, named shengmaxinside C, was obtained as a white amorphous powder, which was considered to be a triterpenoid glycoside due to the positive results with the *Liebermann-Burchard* reaction and *Molish* reagents. Its molecular formula was determined as C_38_H_60_O_12 _according to the positive HRESIMS from the [M-2H_2_O+H]^+^, [M-H_2_O+H]^+^ and [M-H_2_O+Na]^+^ signals at *m/z* 673.3975 (calc. for C_38_H_57_O_10_, 673.3952), 691.4102 (calc. for C_38_H_59_O_11_, 691.4057) and 713.3837 (calc. for C_38_H_58_O_11_Na, 713.3877), respectively, indicating nine degrees of unsauration.

The ^1^H-NMR spectrum (Table 1) showed the presence of cyclopropane methylene groups at *δ*_H_ 0.44 and 1.03 (each 1H, *d*, *J =* 4.0 Hz), six tertiary methyl groups at *δ*_H_ 1.02, 1.18, 1.29, 1.37, 1.49, and 1.74, a secondary methyl at *δ*_H_ 0.95 (1H, *d*, *J =* 6.4 Hz), an acetyl methyl at *δ*_H_ 2.01, one anomeric proton at *δ*_H_ 4.84 (1H, *d*, *J =* 10.4 Hz), four oxygenated methine protons (*δ*_H_ 3.50, 4.21, 4.32, 4.85) and a series of overlapped signals suggesting a cycloartane-type triterpene glycoside. The ^13^C-NMR spectrum (Table 1) showed a total of thirty eight carbon signals due to the aglycon moiety, along with a sugar unit and an acetyl unit. The ^13^C-NMR spectrum exhibited anomeric carbons at *δ*_C_ 107.5. All the above evidence suggested that **3** was a highly oxygenated 9,19-cycloartane triterpene glycoside. Moreover, *δ*_C_ 106.7 indicated **3** to be a hydroshengmanol type saponin [26].

**Figure 2:**
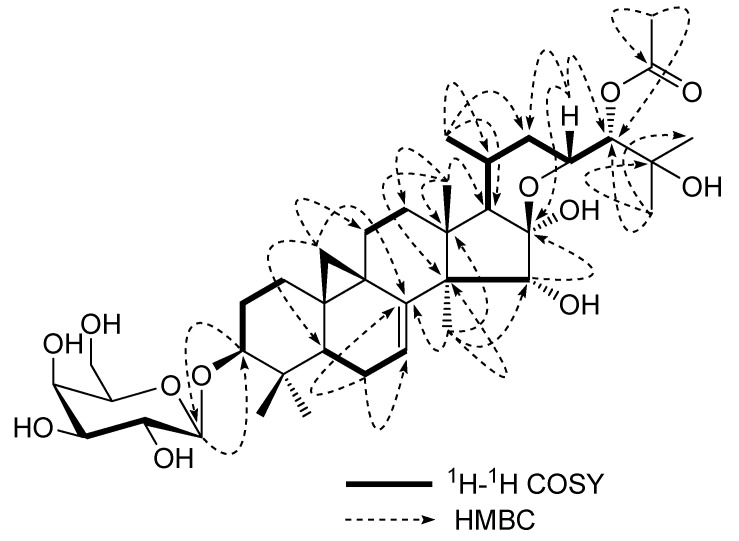
Key HMBC and ^1^H-^1^H COSY correlations of **3**.

After acid hydrolysis and derivatization as alditol acetates, the gas chromatography (GC) analysis revealed the presence of D-galacose. This was further confirmed by its NMR data [16], and the galactose linkage was assigned as *β* form on the basis of the anomeric proton coupling constant at *δ*_H_ 4.84 (1H, *d*, *J =* 10.4 Hz). Three further signals at *δ*_H_ 4.32 (1H, *d*, *J =* 10.4 Hz), 4.85 (1H, *d*, *J =* 2.0 Hz) and 4.21 (*t*, *J =* 8.8 Hz) in the region of the aglycon moiety suggested the presence of three additional oxygen-bearing carbons. This hypothesis was confirmed by the HMBC spectrum, which showed cross-peaks between the proton signal at *δ*_H_ 4.32 (1H, *d*, *J =* 10.4 Hz) with C-14, C-16, C-13 and C-17, between the proton signal at *δ*_H_ 4.85 (1H, *d*, *J =* 2.0 Hz) with C-23 and C-25, and between the proton signal at *δ*_H_ 4.21 (1H, *t*, *J =* 8.8 Hz) with C-22 and C-24, thus unambiguously identifying the oxygen-bearing carbons as C-15, C-23, and C-24. In the HMBC spectrum, significant correlations between *δ*_H_ 4.84(H-1') and 88.4(C-3) suggested that the galactopyranosyl was located at the C-3 position. Moreover, the long-range correlations between H-24 (*δ*_H_ 4.85) with an acetyl carbon (*δ*_C_ 170.7) indicated that the acetyl unit locating at C-24. Other key long-range correlations were found for H-1/C-3, H-19/C-19, 23-H/C-22 and C-24, H-24/C-25 (Figure 2).

According to the literature, the configuration of C-24 is *R* when C-16 chemical shift in the ^13^C-NMR spectrum should be 102.9~103.7, while for *S* it appears to be 106.1~106.8 [19] In the case of **3**, the C-16 chemical shift is 106.7. The ^1^H- and ^13^C-NMR spectrum of **3** were similar to those of 7,8-didehydro-24*S-O*-acetylhydroshengmanol-3-*O*-xyloside [27], respectively, except for the sugar moiety (Table 1). Thus, compound **3** was elucidated as 7,8-didehydro-24*S-O*-acetylhydroshengmanol-3-*O-β*-D-galactopyranoside.

**Table 1:** NMR data for **1-3** in pyridine-*d*_5 _(*J* in Hz).

H/C	1		2		3	
1	1.25 (*m*),1.80 (*m*)	30.3	1.24 (*m*), 1.77 (*m*)	30.4	1.25 (*m*), 1.60 (*m*)	30.3
2	2.43 (*m*),1.94 (*m*)	29.5	2.43 (*dd*,13.2, 4.0), 1.96 (*m*)	29.5	2.42 (*m*), 1.93 (*m*)	29.5
3	3.51 (*dd,* 11.6, 4.0)	88.4	3.52 (*dd*, 11.6, 4.0)	88.5	3.50 (*dd*, 11.6, 4.0)	88.4
4	---	40.4	---	40.4	---	40.4
5	1.25 (*m*)	42.7	1.33 (*m*)	42.8	1.21 (*m*)	42.7
6	1.65 (*m*),1.96 (*m*)	21.2	1.62 (*m*),1.98 (*m*)	21.8	1.60 (*m*), 1.88 (*m*)	21.8
7	6.11 (*d*, 6.4)	114.4	6.07 (*d*, 6.4)	114.3	6.11 (*d*, 6.0)	114.0
8	---	148.0	---	148.1	---	148.4
9	---	21.8	---	21.2	---	21.2
10	---	28.2	---	28.2	---	28.2
11	1.13 (*m*), 2.18 (*overlapping*)	25.6	1.11 (*m*), 2.08 (*overlapping*)	25.5	1.12 (*m*), 2.12 (*overlapping*)	25.6
12	1.66 (*m*),1.83 (*m*)	34.2	1.66 (*m*),1.71 (*m*)	34.0	1.63 (*m*),1.75 (*m*)	34.2
13	---	41.1	---	41.1	---	40.2
14	---	50.8	---	50.8	---	50.0
15	4.53 (*d*, 7.6)	78.4	4.51 (*d*, 7.6)	78.6	4.32 (*d*, 10.4)	80.8
16	---	112.8	---	112.5	---	106.7
17	1.71 (*m*)	60.5	1.72 (*m*)	60.7	1.49 (*m*)	61.2
18	1.17 (*s*)	21.6	1.17 (*s*)	21.6	1.18 (*s*)	22.1
19	0.47 (*d*, 3.6), 1.06 (*d*, 3.6)	28.2	0.44 (*d*, 3.6), 1.06 (*d*, 3.6)	28.4	0.44 (*d*, 4.0), 1.03 (*d*,4.0)	28.3
20	1.70 (*m*)	23.0	1.68 (*m*)	23.4	1.74 (*m*)	25.8
21	0.96 (*d*,5.6)	19.6	0.97 (*d*,5.6)	19.7	0.95 (*d*,6.4)	20.6
22	1.60 (*m*), 2.0 (*m*)	30.5	1.97(*m*), 2.66 (*t*, 22.0,12.0)	29.6	1.57 (*m*), 1.90 (*m*)	33.9
23	4.6 (*ddd*, 2.0, 4.4, 9.2)	73.3	4.62 (*ddd*, 2.0, 4.4, 9.2)	73.9	4.21 (*dd*, 2.0, 8.8)	72.8
24	3.77 (*d*, 4.4)	84.1	3.72 (*d*, 4.4)	84.1	4.85 (*d*, 2.0)	80.3
25	---	79.8	---	68.6	---	75.5
26	1.64 (*s*)	24.6	1.41 (*s*)	30.7	1.49 (*s*)	32.8
27	1.53 (*s*)	23.2	1.33 (*s*)	25.9	1.74 (*s*)	27.2
28	1.47 (*s*)	18.5	1.27 (*s*)	18.5	1.37 (*s*)	18.8
29	1.33 (*s*)	25.9	1.28 (*s*)	26.0	1.29 (*s*)	25.8
30	1.04 (*s*)	14.3	1.03 (*s*)	14.3	1.02 (*s*)	14.3
1'	4.89 (*d*, 7.6)	107.5	4.88 (*d*,8.0)	107.5	4.84 (*d*, 10.4)	107.5
2'	4.49 (*m*)	73.2	4.47 (*m*)	73.2	4.46 (*dd*, 9.2, 4.0)	73.2
3'	4.17 (*dd*, 9.6, 3.2)	75.5	4.17 (*dd*, 9.2,3.2)	75.5	4.16 (*dd*, 9.4, 3.4)	75.2
4'	4.60 (*overlapping*)	70.3	4.59 (*overlapping*)	70.3	4.59 (*d,* 3.2)	70.3
5'	4.09 (*t*, 12.4,8.4)	76.9	4.08 (*t*, 12.4, 6.0)	76.8	4.08 (*t*, 10.0, 6.2)	76.8
6'	4.48 (*overlapping*), 4.50 (*overlapping*)	62.5	4.47 (*overlapping*), 4.48 (*overlapping*)	62.5	4.42 (*t*, 9.2, 4.0), 4.46(*t*,9.2 ,4.0)	62.5
-CO CH_3_	---	169.8	---	---	---	170.7
-CO CH_3_	2.01(*s*)	22.6	---	---	2.01 (*s*)	21.0

## 3. Experimental Section

### 3.1. General

The optical rotations were recorded on a Perkin-Elmer 341 polarimeter. IR spectra were taken on a Shimadzu FTIR-8400 S. The NMR spectra were recorded on a Bruker DPX 400 instrument (400 MHz for ^1^H-NMR and 100 MHz for ^13^C-NMR). Samples were prepared in pyridine-d_5_ with TMS as an internal standard and coupling constants *J* are given in Hz. The UV spectra were recorded on a Shimadzu UV-1601 instrument and GC analysis was carried out on an Agilent HP 6890N gas chromatograph using an HP-5 capillary column. The HRESIMS was determined on an IonSpec Ultima 7.0 T FTICR. Preparative HPLC (Waters, Delta 600-2487) was performed on Hypersil-ODS II (10 μm, 20×300 mm, Yilite, Da Lian, China). Column chromatography was performed with silica gel (200-300 mesh, Qingdao Haiyang Chemical Group Co. Ltd, Qingdao, P. R. China), ODS-A (120A, 50μm, YMC Co.) and Sephadex LH-20 (25-100 μm, Pharmacia). Analytical TLC spots were detected on silica gel 60 F254 (Merck, Germany) by spraying with 10% ethanolic H_2_SO_4_ reagent followed by heating. 

### 3.2. Plant Material

Root of *C.* *simplex* was collected in Heilong Jiang province of China, on September 2009, and identified by Prof. Wang (Heilongjiang University of Chinese Medicine). The voucher specimen (20090065) was deposited at the Herbarium of Heilongjiang University of Chinese Medicine, Harbin, China.

### 3.3. Extraction and Isolation

The roots of *C. simplex* (2.6 kg) was extracted under reflux conditions with 75% ethanol (3L×3×2 h each). The ethanolic solution was concentrated *in vacuo* to yield a syrup-like extract (225 g), which was dissolved in H_2_O (1500 mL) and then partitioned with different solvents to give petroleum ether–soluble (7.6 g), ethyl acetate-soluble (75 g) and *n*-butyl alcohol-soluble (19g) portions. The ethyl acetate-soluble portion was subjected to silica gel column chromatography (CHCl_3_/MeOH, 20:1→1:1) to afford Fractions A-H. Fraction D (6 g) was re-chromatographed on silica gel (200-300 mesh, 150 g), eluted with CHCl_3_-MeOH (20:1) as solvent, to afford three sub-fractions. Sub-fraction D_2_ (3.6 g) was further separated by ODS (MeOH/H_2_O, 6:4→9:1) to afford five fractions. Fraction D_2-3_ was followed by Sephadex LH-20 and purified by preparative HPLC with MeOH/H_2_O 7:3 to afford compound 1 (23 mg). Fraction D_2-4_ was purified by preparative HPLC with MeOH/H_2_O 6:4 to furnish **2** (28 mg). Fraction E (3.3 g) was further chromatographed on OSD (MeOH/H_2_O, 1:1→9:1) to afford three fractions. Compound **3** (23 mg) was purified from the Fraction E_2_ by repeated ODS and HPLC methods.

*Shengmaxinside A* (**1**). Colorless needles; [α]^25^_D_: +0.02 (MeOH); IR (KBr): 3431.13, 3423.41, 2956.67, 2937.38, 2871.81, 1730.03, 1367.44, 1240.14, 1151.42, 1070.42, 1058.85, 1043.42, 975.91 cm^−1^; HRESIMS from the [M-H_2_O+H]^+^ and [M+Na]^+^ signals at *m/z* 673.3964 (calc. for C_38_H_57_O_10_, 673.3952) and 713.3894 (calc. for C_38_H_58_O_11_Na, 713.3877); ^1^H-NMR and ^13^C-NMR data are shown in Table 1.

*Shengmaxinside B* (**2**). Colorless needles; [α]^25^_D_: +0.03 (MeOH); IR (KBr): 3431.13, 3421.48, 2960.53, 2931.6, 2871.81, 2358.78, 2341.42, 2331.78, 1155.28, 1056.92, 987.49, 977.84 cm^−1^; HRESIMS from the [M-H_2_O+H]^+^ and [M+Na]^+^ signals at *m/z* 631.3887 (calc. for C_36_H_55_O_9_ 631.3846) and 671.3804 (calc. for C_36_H_56_O_10_Na 671.3771); ^1^H-NMR and ^13^C-NMR data are shown in Table 1.

*Shengmaxinside C* (**3**). White amorphous powder; [α]^25^_D_: +0.02 (MeOH); IR (KBr): 3411.84, 2952.81, 2935.46, 1718.46, 1379.01, 1244.00, 1163.00, 1151.42, 1062.7, 1031.85, 981.7 cm^-1^; HRESIMS from the [M-2H_2_O+H]^+^, [M-H_2_O+H]^+^ and [M-H_2_O+Na]^+^ signals at *m/z* 673.3975 (calc. for C_38_H_57_O_10_, 673.3952), 691.4102 (calc. for C_38_H_59_O_11_, 691.4057) and 713.3837 (calc. for C_38_H_58_O_11_Na, 713.3877); ^1^H-NMR and ^13^C-NMR data are shown in Table 1. 

### 3.4. Acid hydrolysis

Acid hydrolysis was performed by a previously described method [28]. For this purpose, each compound (10 mg) was heated in an ampule with aqueous 12% HCl (5 mL) at 90 °C for 2h. The aglycone was extracted with chloroform, and each aqueous residue was adjusted to pH 7.0 with 12% NaOH and reduced with NaBH_4_ (40 mg), followed by acidification with dilute CH_3_COOH, and then co-distilled with pure CH_3_OH to remove excess boric acid. The reduced sugars were acetylated with 1:1 pyridine-Ac_2_O in a boiling water bath for 2 h to give the corresponding alditol acetates, which were analyzed by GLC on a HP 6890 N gas chromatograph (Agilent) equipped with a flame ionization detector FID) using N_2_ as carrier gas. The instrument was fitted with a HP-5 capillary column (30 m×0.32mm×0.25 μm). The injector temperature was set at 250 °C and the column temperature program was as follows: the initial temperature of 120 °C was increased by 3°/min to the final temperature of 210 °C, then was held 4 min. The detector temperature was set at 300 °C. The standard monosaccharides were subjected to the same reaction and GC analysis under the same conditions (D-galacose, t_R_, 30.8 min)

## 4. Conclusions

It has been reported that 9,19-cyclolanostane triterpene glycosides exhibit varied biological activities, including antiosteoporosis, antitumor, anti-complement, antioxidant and anti-inflammatory effects [23,24,25]. As a part of our chemical investigation on *C. simplex*, three new cycloartenol triterpene saponins with galactopyranosyl moieties, shengmaxinsides A-C, were isolated. Their structures were established on the basis of spectroscopic analysis and chemical evidence. Their biological activities will be further researched in our laboratory. 
